# Disappearing Scales in Carps: Re-Visiting Kirpichnikov's Model on the Genetics of Scale Pattern Formation

**DOI:** 10.1371/journal.pone.0083327

**Published:** 2013-12-30

**Authors:** Laura Casas, Réka Szűcs, Shubha Vij, Chin Heng Goh, Purushothaman Kathiresan, Sándor Németh, Zsigmond Jeney, Miklós Bercsényi, László Orbán

**Affiliations:** 1 Reproductive Genomics Group, Strategic Research Program, Temasek Life Sciences Laboratory, Singapore, Singapore; 2 Department of Animal Sciences and Animal Husbandry, Georgikon Faculty, University of Pannonia, Keszthely, Hungary; 3 Fish Facility, Temasek Life Sciences Laboratory, Singapore, Singapore; 4 Research Institute for Fisheries, Aquaculture and Irrigation, Szarvas, Hungary; 5 Department of Biological Sciences, National University of Singapore, Singapore, Singapore; University of Sheffield, United Kingdom

## Abstract

The body of most fishes is fully covered by scales that typically form tight, partially overlapping rows. While some of the genes controlling the formation and growth of fish scales have been studied, very little is known about the genetic mechanisms regulating scale pattern formation. Although the existence of two genes with two pairs of alleles (S&s and N&n) regulating scale coverage in cyprinids has been predicted by Kirpichnikov and colleagues nearly eighty years ago, their identity was unknown until recently. In 2009, the ‘S’ gene was found to be a paralog of fibroblast growth factor receptor 1, *fgfr1a1*, while the second gene called ‘N’ has not yet been identified. We re-visited the original model of Kirpichnikov that proposed four major scale pattern types and observed a high degree of variation within the so-called scattered phenotype due to which this group was divided into two sub-types: classical mirror and irregular. We also analyzed the survival rates of offspring groups and found a distinct difference between Asian and European crosses. Whereas nude × nude crosses involving at least one parent of Asian origin or hybrid with Asian parent(s) showed the 25% early lethality predicted by Kirpichnikov (due to the lethality of the NN genotype), those with two Hungarian nude parents did not. We further extended Kirpichnikov's work by correlating changes in phenotype (scale-pattern) to the deformations of fins and losses of pharyngeal teeth. We observed phenotypic changes which were not restricted to nudes, as described by Kirpichnikov, but were also present in mirrors (and presumably in linears as well; not analyzed in detail here). We propose that the gradation of phenotypes observed within the scattered group is caused by a gradually decreasing level of signaling (a dose-dependent effect) probably due to a concerted action of multiple pathways involved in scale formation.

## Introduction

Cyprinid teleosts account for over 30% of worldwide aquaculture production and according to the FAO, common carp (*Cyprinus carpio L.*) is the species with the third highest production today (http://www.fao.org/fi/default.asp). Common carp was probably the earliest domesticated fish species for alimentary purposes, with records of ancient Chinese documents showing that cultivation of common carp in China began in the twelfth century BC [1], [2], [3]. In Europe, common carp was first domesticated by the Romans before the sixth century [1], [2], [3], [4].

Today, common carp is divided into at least two subspecies: the separation of Central-Asian/European (*C. carpio carpio*) and East-Asian subspecies (*C. carpio haematopterus*) is well supported by microsatellite and mitochondrial genetic data [5], [6], [7], [8]. In addition, the existence of a potential third subspecies (*C. c. rubrofuscus or C. c. viridiviolaceus*) is possible, but not confirmed based on the genotypes [6]. Earlier, a Central-Asian subspecies (*C. c. aralensis*) was proposed by Kirpichnikov [9]. Subsequent studies [5], [6], [10] have demonstrated that the European and Central-Asian forms of common carp are actually quite closely related, with the latter comprising a subset of the genetic diversity of the former. The authors eventually classified both European and Central-Asian carp as subspecies *carpio*. Based on the analysis of mtDNA sequences, Froufe and colleagues [11] concluded that the European common carps were likely introduced from Asia.

The domestication of common carp led to the emergence of different varieties and among them, various scalation patterns. These variants, characterized by the reduction of the scale coverage, have been favoured as they were easier to de-scale for cooking [12]. According to Kirpichnikov [9], [13], [14], the main scalation types of common carp are: scaled, linear, scattered and nude (Table 1; Supplementary File S1A–D). In scaled carps, the whole body is covered with regularly arranged, partially overlapping scales of very similar size (wild-type). In linear carps, there is a clearly defined line of a uniform set of scales below the dorsal fin and over the lateral line and also, in some cases, a lesser number of scales can be found dispersed over the body surface. The scattered phenotype is characterized by a (near) complete line of scales below the dorsal fin and a similar set of scales above the belly (usually more incomplete than the line of scales below the dorsal fin). In addition, there may be few scales scattered over the body surface. On the other hand, individuals are classified as nude if scales are either totally absent or very few scales are present (can be randomly distributed, but can also be seen as an incomplete line of scales below the dorsal fin). This phenotype is always accompanied by a reduced number of pharyngeal teeth and often by fin defects as well. In addition to the above phenotypes, several additional varieties, including those that would be described in this manuscript have also been reported [13], [15], [16], but they have mostly been regarded as deviations and therefore, have not been included in the genetic model (see below).

**Table 1:** Punnett square showing the expected distribution of offspring scale pattern phenotypes from a cross between two common carp brooders heterozygous for both loci involved in scale formation based on Kirpichnikov's system.

Genotypes	S;n	s;n	S;N	s;N
S;n	Scaled	Scaled	Linear	Linear
s;n	Scaled	Scattered	Linear	Nude
S;N	Linear	Linear	Lethal	Lethal
s;N	Linear	Nude	Lethal	Lethal

The distribution of scales over the body of cyprinids is genetically determined. Rudzinsky [17], [18] was the first to point out that the fully scaled trait is dominant over the mirror one. Based on data obtained by remarkably simple tools, such as survival rates and phenotypic analysis of individuals grown in ponds, Kirpichnikov and colleagues proposed that the process is regulated by up to four genes [19], and later they refined their theory to a ‘two genes – four alleles’ type model for the inheritance of scale pattern in common carp [13], [14]. According to their model, scaled fish are of SS/nn or Ss/nn genotype, linears are SS/Nn or Ss/Nn, scattered carps are ss/nn, while nudes (or ‘leathers’) are ssNn (for review see: [13], [14]; Supplementary File S1). Moreover, NN results in lethality in any combination with ss, SS or Ss. Accordingly, as illustrated in the Punnett square (Table 1), a theoretical dihybrid cross involving two brooders heterozygous for both loci should produce five phenotypes with the following expected frequencies: scaled (3/16); linear (6/16); scattered (1/16), lethal (4/16) and nude (2/16).

Over the next decades, the majority of textbooks took over the model and it became the most well-known example for two-genic inheritance in fish genetics (see e.g. [20], [21]). Although some of the crosses were repeated subsequently and yielded data similar to the original ones (see e.g. [22], [23]), according to our knowledge, nobody has re-visited the issue by performing a systematic analysis of a larger set of crosses. Recently, two findings motivated us to reconsider the model. The first result was that nude × nude common carp crosses performed at one of the Hungarian fish farms repeatedly failed to show either the 25% lethality, or the 25% of scattered phenotypes [15] expected on the basis of the Kirpichnikov model [13], [14]. The second was the discovery of a “mirror” variant in zebrafish and the identification of the mutant gene responsible for this phenotype: one of the paralogs of fibroblast growth factor receptor 1, *fgfr1a* in zebrafish and *fgfr1a1* in common carp [24]. In other words, this is the ‘s’ gene predicted earlier based on data from common carp by Kirpichnikov and his team [13], [14], [19], [25]. Though this gene has an essential function during embryonic development, the presence of another paralog in both zebrafish and the common carp ensures a milder, viable phenotype. This is in contrast to Japanese medaka (*Oryzias latipes*), wherein a single copy is present. The homozygous mutant medaka embryos thus show a drastic phenotype with a severe body loss and only the head being able to develop [26]. Mutation of another FGF pathway gene, *fgf20a*, results in feather loss in chicken [27]. The FGF pathway also plays an important role in the formation of median fin fold, the precursor of dorsal fin [28]; paired fins [29] and lateral line in the zebrafish (reviews: [30], [31], [32]), as well as fin regeneration (reviews: [33], [34]). In addition to the FGF signalling pathway, mutation of gene(s) in the ectodysplasin (Eda) pathway have been shown to affect the development of scales in medaka [35], scales along with additional skeletal and dental structures such as skull, fins and teeth in zebrafish [36], [37], dermal plate formation in stickleback [38], hair and teeth in mouse and humans [39], [40], [41] as well as chicken feathers [42], [43]. In addition, retinoic acid (RA) signalling has also been implicated in influencing teeth numbers in zebrafish [44] and mutation in downstream effector of Wnt signaling (*lef1*) pathway has also been shown to effect the teeth numbers and gill rakers in zebrafish [45]. Thus, multiple pathways have been shown to affect the scale pattern as well as teeth number across various fish and mammalian species. These discoveries will pave the way for a more informed search for the second member, the so-called ‘N’ gene, whose mutation in an ‘ss’ individual would cause either complete scale loss in ‘ssNn’ heterozygotes or lethality in ‘ssNN’ homozygotes.

In this manuscript, we revisited Kirpichnikov's model of scale pattern inheritance and described the ratio of scale pattern phenotypes in offspring groups originating from crosses involving brooders with partial or full loss of scale sets. We also observed a gradation in the number of scales, teeth and fins that extended well outside of those individuals showing the nude phenotype. Based on these data, we propose a model that could help to focus future searches aimed for the identification of the mysterious ‘N’ gene.

## Materials and Methods

### Ethics statement

All experiments performed in Singapore were approved by the Temasek Life Sciences Laboratory Institutional Animal Care and Use Committee (approval ID: TLL (F)-10-003). The experiments in Hungary have been carried out under the permits of Food-chain Safety and Animal Health Directorate of Zala County Agricultural and Technical Management Office (No: 29.1/318/8/2008) and the Food-chain Safety and Animal Health Directorate of Governmental Office of Zala County (No: 1-100/2258-002/2012).

### Brooders

Carps were maintained under standard conditions of fish husbandry unless indicated otherwise below. For the crosses performed in Hungary, common carp brooders (males and females) were selected from the following sources: scaled carp - Amur wild type carp, and Tata common carp from the live cyprinid collection of HAKI (Szarvas, Hungary; offspring used for the comparative analysis of fin and tooth losses only); mirror carps - Line No2 from HAKI; linear carps from Tiszaker fish farm (Kőröstarcsa, Hungary) and nude carps from Béke Fish Farm (Hajdúböszörmény, Hungary).

For the crosses performed in Singapore, a nude male carp originating from the Béke Fish Farm was shipped from Hungary to Singapore and used as a father for a large number of crosses. In addition to that, koi carps of the four major and some minor scale pattern types were purchased from Qian Hu Fish Farm (Singapore), and used as brooders.

### Artificial propagation

The brooders were prepared for artificial propagation by hypophysation [4]. Small batches of eggs (ca. 50 g) from each female were fertilized by 2 ml of fresh milt collected earlier from the chosen male(s). For the crosses performed in Hungary, two minutes after fertilization, the eggs were stacked onto a tulle netting that was stretched onto a metal frame. This provided easy and accurate tracking of embryonic development, as fertilization rate and hatching percentage were calculated by counting the live or dead eggs using digital photos of the eggs stacked to the net. For the crosses performed in Singapore, the stickiness of fertilized eggs was first removed through a treatment with Woynarovich solution [46] and later they were placed into traditional McDonald jars and they were hatched there. Survival rates were calculated by removing a random sample of eggs and counting live vs. dead individuals under a stereo microscope.

### Hatching, larviculture and phenotyping

**Figure 1:**
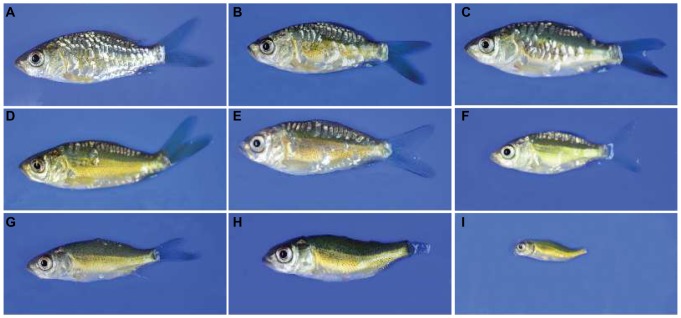
Variations observed in Kirpichnikov's “scattered” scale pattern and nude phenotypes. The scattered phenotype can be further divided into two sub-types: irregular (panels A–C) and classical mirror (D–F). The nude phenotype can also be divided into three sub-types: nude1 (G), nude2 (H) and nude3 (I). (These three sub-types were not separated during the analysis of phenotypes.).

Fry were hatched out in separate tanks in order to avoid potential mixing of different families. Feeding of fry started on the 3rd day after hatching by micropellet feed (Rescue, Japan) in Singapore or artemia in Hungary. From the end of the second week, live food was gradually replaced with dry pelleted feed over a week's transition (Hungary). In Singapore, families were divided into several 9 L tanks at the density of 50–60 individuals per liter, whereas slow growing individuals, especially those showing special swimming pattern(s) were separated from the rest during the first week after fertilization and grown in smaller tanks at <20/L density. At about two months post-fertilization (mpf), the fish were transferred into 200 L tanks connected to a recirculation system at 200 individuals per tank density. As they grew, their numbers were reduced systematically by random removal to keep the density below 10 kg/m^3^. In Hungary, the fry were placed into 20 L tanks and fed live brine shrimp and Perla larval feeds (Skretting; Perla Larva; Italy.). Later they were transferred to 60 L followed by 300 L tanks and were fed Skretting Classic 1P–3P feeds. The families were reared for four months so that the scale pattern could be clearly identified. At this time point, for the first two crosses performed in Singapore (#1nu.nu&#2nu.nu; Table 2; Supplementary Files S2&S3), classification was performed directly through visual observation of the fish, whereas for the remaining Singaporean crosses and all crosses performed in Hungary, fingerlings were individually photographed from both sides and scalation was assessed based on the photos. Phenotypic analysis was performed by assessing the scale patterns based on a classification (see Supplementary File S4) that is a modified version of Kirpichnikov's [13], [14], as our classification contains a total of five categories instead of the four used earlier. We have retained three of the four major scale patterns, namely, scaled, linear, and nude (Supplementary File S1). In addition, we have divided Kirpichnikov's ‘scattered’ category into two sub-categories: irregular (mirror with additional scales that are typically larger in size and cover most of the body surface often without partial overlaps) and mirror (Fig. 1; for detailed descriptions see Supplementary File S4). Phenotype frequencies within the families as percentage were compared to the expected values calculated from the Kirpichnikov model.

**Table 2:** Distribution of scale phenotypes from 19 different crosses.

No	Cross*	Cross Type	Location*	M_Orig	Fe_Orig	M_ID	Fe_ID	F1_No	Expected %					Observed %				
									Scaled	Linear	Scattered	Nude	Lethal	Scaled	Linear	Irreg	Mirr	Nude
1	1nu.nu	nude × nude	SIN	Hun	Koi	CcB01**	CcB02	161			25%	50%	25%	0.0%	0.0%	15.5%	25.5%	59.0%
2	2nu.nu	nude × nude	SIN	Koi	Koi	CcB03	CcB02	92			25%	50%	25%	1.1%	0.0%	15.2%	25.0%	58.7%
**3**	**26nu.nu**	**nude × nude**	**HUN**	**Hun**	**Hun**	**CcB04**	**CcB05**	**208**			**25%**	**50%**	**25%**	**0.0%**	**0.0%**	**0.0%**	**13.0%**	**87.0%**
4	35nu.nu	nude × nude	SIN	Hun	Koi	CcB01	CcB06	218			25%	50%	25%	0.0%	0.0%	53.2%	2.3%	44.5%
5	38nu.nu	nude × nude	SIN	Hun	F1hyb	CcB01	CcB07	110			25%	50%	25%	0.0%	0.0%	55.5%	20.0%	24.5%
6	41nu.nu	nude × nude	SIN	Hun	Koi	CcB01	CcB08	253			25%	50%	25%	0.0%	0.0%	35.6%	26.1%	38.3%
**7**	**21nu.mi**	**nude × mirror**	**HUN**	**Hun**	**Hun**	**CcB04**	**CcB09**	**186**			**50%**	**50%**		**0.0%**	**1.1%**	**0.0%**	**96.2%**	**2.7%**
**8**	**25mi.nu**	**nude × mirror**	**HUN**	**Hun**	**Hun**	**CcB10**	**CcB05**	**233**			**50%**	**50%**		**0.0%**	**0.0%**	**0.0%**	**100.0%**	**0.0%**
9	32nu.mi	nude × mirror	SIN	Hun	F1Hyb	CcB01	CcB11	268			50%	50%		0.0%	0.0%	57.5%	18.2%	24.3%
10	36mi.nu	nude × mirror	SIN	F1hyb	Koi	CcB12	CcB06	177			50%	50%		0.0%	0.0%	73.4%	1.7%	24.9%
11	34nu.ir	nude × irreg.	SIN	Hun	F1Hyb	CcB01	CcB13	414			50%	50%		0.0%	0.0%	76.1%	18.4%	5.5%
12	39ir.nu	nude × irreg.	SIN	F1hyb	F1hyb	CcB14	CcB07	54			50%	50%		0.0%	0.0%	44.5%	22.2%	33.3%
13	40nu.ir	nude × irreg.	SIN	Hun	F1hyb	CcB01	CcB15	114			50%	50%		0.0%	0.0%	52.6%	34.2%	13.2%
14	33mi.ir	mirror × irreg.	SIN	F1hyb	F1hyb	CcB12	CcB13	304			100%			0.0%	0.0%	86.2%	13.2%	0.6%
15	37mi.ir	mirror × irreg.	SIN	F1hyb	F1hyb	CcB12	CcB14	236			100%			0.0%	0.0%	64.8%	35.2%	0.0%
**16**	**22nu.li**	**linear × nude**	**HUN**	**Hun**	**Hun**	**CcB04**	**CcB15**	**118**	**12.5 or 25%** ***	**25 or 50%**	**0 or 12.5%**	**0 or 25%**	**25%**	**0.0%**	**2.5%**	**0.0%**	**88.2%**	**9.3%**
**17**	**27li.nu**	**linear × nude**	**HUN**	**Hun**	**Hun**	**CcB16**	**CcB05**	**289**	**12.5 or 25%** ***	**25 or 50%**	**0 or 12.5%**	**0 or 25%**	**25%**	**0.0%**	**1.0%**	**0.0%**	**98.6%**	**0.3%**
**18**	**9mi.li**	**linear × mirror**	**HUN**	**Hun**	**Hun**	**CcB17**	**CcB18**	**52**	**25 or 25%** ***	**25 or 50%**	**25 or 50%**	**0 or 25%**		**0.0%**	**67.3%**	**1.9%**	**23.1%**	**7.7%**
**19**	**23mi.li**	**linear × mirror**	**HUN**	**Hun**	**Hun**	**CcB10**	**CcB15**	**204**	**25 or 50%** ***	**25 or 50%**	**25 or 50%**	**0 or 25%**		**0.5%**	**32.8%**	**0.0%**	**66.7%**	**0.0%**

Abbreviations: M – male; Fe –female; nu – nude; mi – mirror; irreg – irregular; and li – linear.

*Crosses produced in Hungary are displayed in bold throughout the table.

**For information on brooders, see Supplementary Files S2 & S3;

***Depending on the genotype of the linear parent: SSNn or SsNn.

### Isolation of pharyngeal teeth

For the isolation of pharyngeal teeth, individuals were culled by placing them into 2% ethyl 3-aminobenzoate methanesulfonate salt (MS222; Sigma-Aldrich, St. Louis, MO, USA) for 15 minutes. Then, their head portion was cut off at the distal end of the operculum and submerged in 4% potassium hydroxide to dissolve the soft parts. After 2–3 days, the pharyngeal teeth were picked from the remaining mass of tissue and thoroughly washed in water and dried. The number of teeth was counted under a Leica M125 stereomicroscope and the photographs were taken with a Leica MZ 10 F stereomicroscope fitted with a Nikon DXM 1200 F camera.

## Results

### The scale pattern distribution in the offspring of carp brooders with partial or full scale loss does not always follow the Kirpichnikov system

We generated 19 different crosses with 18 common carp brooders (see Table 2 and Supplementary Files S2&S3 for details) and analyzed the scale pattern phenotypes of the resulting offspring. All of the crosses involved at least one brooder with reduced scale pattern: 14 were between the classical scalation types (i.e. linear, mirror and nude), whereas in the remaining five, one of the parents showed the ‘irregular’ scale pattern (see Supplementary File S4 for detailed description of phenotypes). When classified according to the origin of the parents, eight crosses involved partners originating from the same subspecies (Hungarian × Hungarian or koi × koi), three of them were between the two subspecies and the remaining eight involved one or two F1 hybrids from a cross between the two subspecies.

In several cases, we found substantial deviation from the ratios predicted based on Kirpichnikov's model [13], [14]. In the five nude × nude crosses involving at least one koi parent (and four from the same Hungarian male; CcB01), 33% mirrors and 67% nudes were expected after the initial loss of one quarter of the offspring (due to the inviability of NN genotypes). The proportion of nudes in the first two crosses was close to the expected value (#1nu.nu – 59%; #2nu.nu – 58.7%), whereas for the remaining three it was much lower (#35nu.nu – 44.5%; #41nu.nu – 38.3% and #38nu.nu – 24.5% only). The rest of the individuals showed a scattered (i.e. either mirror or irregular) phenotype in all five crosses. Interestingly, a substantial proportion (15–55%) of irregular offspring individuals appeared in all crosses (Table 2). In the only cross between two Hungarian nudes (#26nu.nu), the proportion of nudes increased to 87% (expected: 67%) presumably due to the lack of lethality and the remaining 13% of the offspring were all mirrors (Table 2).

Altogether, seven nude × scattered type crosses were analyzed. Four of them involved a nude and a mirror parent (two from Hungary and two from Singapore), whereas the remaining three were from nude × irregular parental combinations (all from Singapore). None of these seven crosses produced the 50% nudes predicted by Kirpichnikov. The most extreme deviation was shown by the two Hungarian nude × mirror crosses (#21mi.nu & #25nu.mi) that yielded only 2.7% and 0% nudes, respectively (Table 2). In the remaining five crosses generated in Singapore, the proportion of nudes ranged from 5.5% to 33.3% (vs. the expected 50%), whereas the rest of the offspring were either irregulars or mirrors, with the former being in the majority (Table 2).

In the two crosses involving mirror and irregular parents (#33mi.ir and #37mi.ir), only mirror and irregular offspring were expected. Accordingly, no fully scaled or linear individuals were found among the offspring and less than 1% nudes were found in one cross (#33mi.ir), whereas none in the second cross. Among the scattered offspring, the proportion of the new sub-category (irregular) dominated the phenotype list (86% and 65%, respectively). The rest of them were mostly classical mirrors in both cases: 13% and 35%, respectively (Table 2).

Altogether, four crosses with one linear parent were analyzed (all with both parents of Hungarian origin). In the two linear × nude type crosses (i.e. #22li.nu & #27nu.li) maximum 12.5% scattered (i.e. irregular and/or mirror) were expected and 88.1–98.6% were found. On the other hand, no fully scaled offspring were produced in all four crosses (with the exception of 23li.mi, where a single such individual was found), despite the predicted range being 12.5–50%, depending on the parental genotypes (Table 2). The ratio of the nude offspring also did not meet those expected based on the Kirpichnikov system in three of the four crosses: 0.3–9.3% (detected) vs. 0% or 25% (expected; Table 2).

### Lack of the expected 25% lethality among the offspring of Hungarian common carps with nude and linear scale pattern types

**Figure 2:**
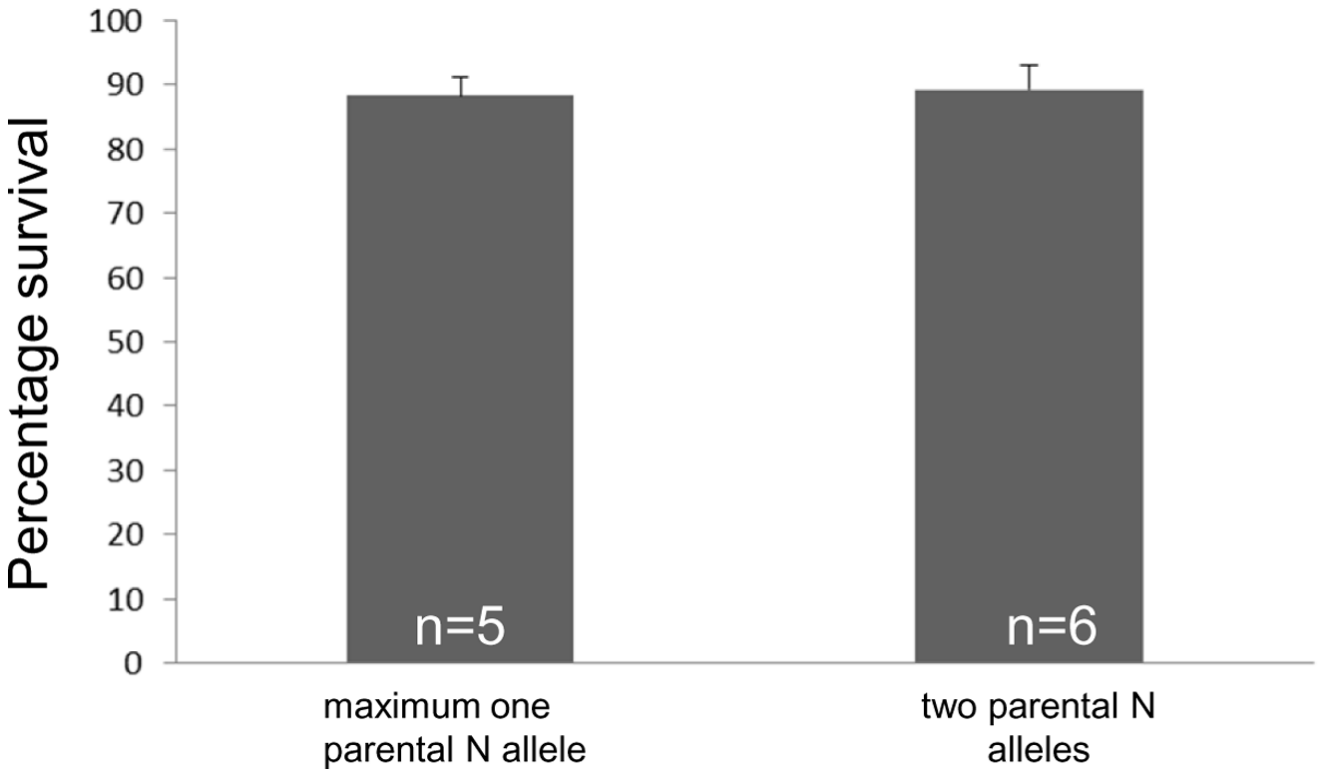
The survival rates from crosses with two nude/linear parents and those with only one were not different. All brooders were of European origin. Survival percentage was measured on seventh day post hatching and calculated as live fry number/total egg number. Three to five technical replicates per sample were counted. The difference between the two groups is not statistically significant (Student's t-test; P = 0.63).

We have determined the survival rates of the offspring either by i) counting fertilized eggs with (viable embryos) or without eye spots (dead eggs) stuck onto nets; or ii) sorting a few hundred embryos randomly removed from the hatching jar under a dissecting scope. Analysis of the survival rates showed the expected 25% lethality (due to the inviability of NN individuals) in all nude × nude crosses performed in Singapore (data not shown), but not among the offspring of Hungarian linear × linear, linear × nude or nude × nude crosses. The combined mean survival rate for these latter three offspring groups was 89.16+/−3.76%, not significantly different from that of those crosses, where no NN offspring individuals could be theoretically produced (88.20+/−2.77%; P = 0.63; Student's t-test; Fig. 2 and Supplementary File S5).

### The deformity/disappearance of fins and gradual decrease in pharyngeal teeth count could be observed in the scattered phenotype, not just the nude one

**Figure 3:**
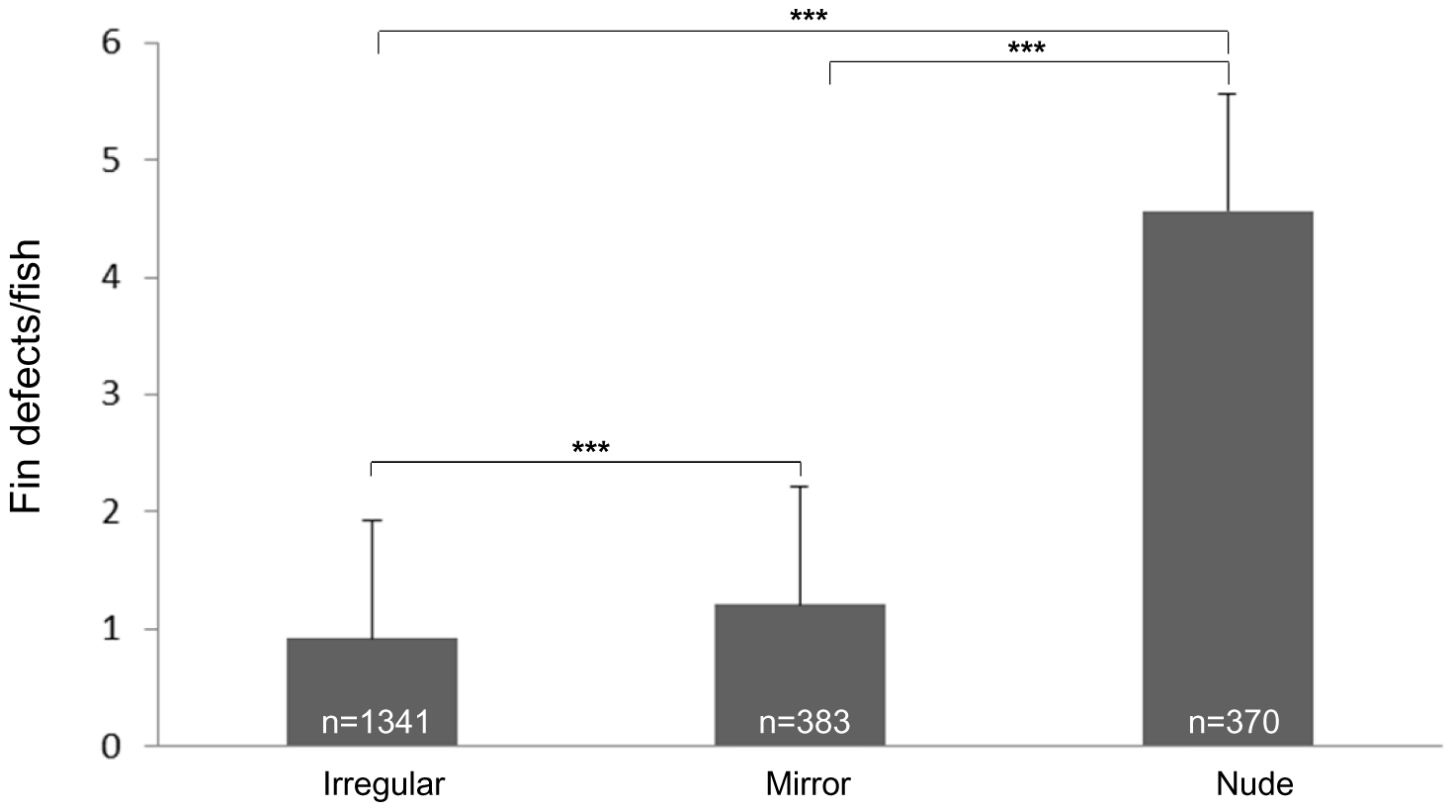
Association between the level of scale loss and fin defects in irregular, mirror and nude phenotypes across nine families. (A) Fin defects were quantified on a per fish basis using an arbirary scale (absence: 1 point, stunting: 0.75 point, reduction: 0.5 point and slight reduction: 0.25 point). The average of fin defects per fish are plotted for each group along with the standard deviation. *** indicates P-value (<0.005) calculated using Student's t-test. Numbers in white at the bottom of the bars indicate the number of individuals analyzed from each scale-pattern type.

We tested potential associations between various levels of scale loss and fin deformity and/or loss in irregular, mirror and nude individuals from nine families originating from crosses involving European and Asian grandparents (#32nu.mi, #33.mi.ir, #34.nu.ir, #35nu.nu, #36mi.nu; #37mi.ir, #38.nu.nu, #40nu.ir & #41nu.nu; Table 2). Fin defects showed a progressive increase with the decrease in the number of scales such that the irregular individuals had the least of these abnormalities in terms of fins being distorted (reduced/stunted) or absent while the nude group had the maximum number of such defects (Fig. 3). Fin defects (including loss) were quantified on a per-fish basis using an arbitrary cummulative scale (absent fin: 1 point; stunted fin: 0.75 point; reduced fin: 0.5 point and slightly reduced fin: 0.25 point). The average fin defects were the highest for nudes (4.5 points) and the lowest for irregulars (<1 point) with mirrors showing 1.2 points of the loss/distortions on an average (Fig. 3). The pairwise differences were significant between each of the pairs tested (P<0.01; Student's t-test).

When sorted according to the fin type affected, the mirrors and nudes showed a similar percentage of reduced/stunted fins across all the fin types with nudes showing a slightly higher percentage of these defects. On the other hand, the percentage of such defects was significantly lesser in the irregular group (Supplementary File S6). The same trend was seen for absence across the different fin types, with the exception of the paired pectoral fins where the irregulars and mirrors had a very marginal percentage of absence (∼2%) while these fins were absent in >50% of nudes.

**Figure 4:**
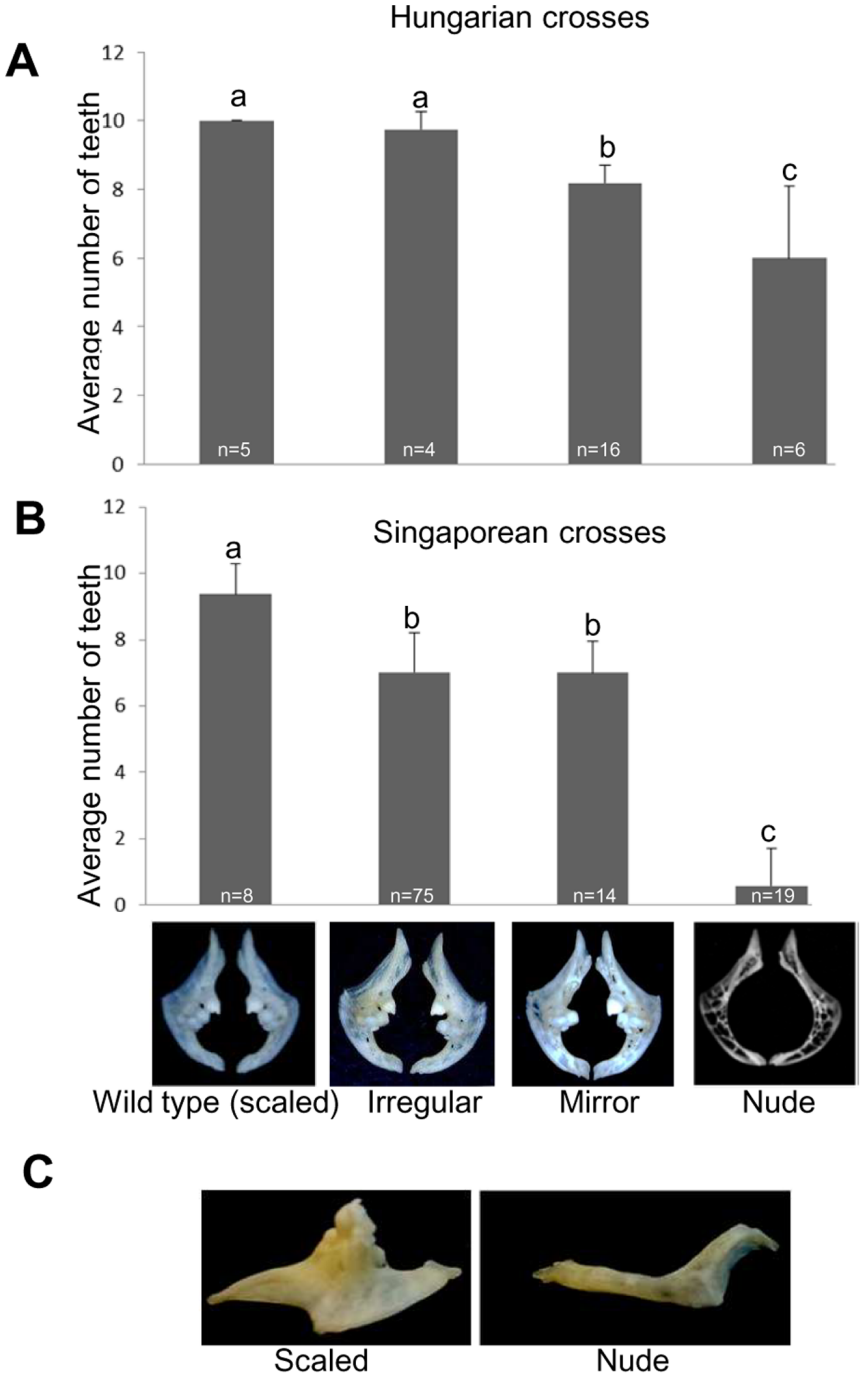
The number of pharyngeal teeth gradually decreases with the reduction of scale coverage from completely scaled to nudes. The average number of teeth is shown for the four scale-pattern phenotypes (scaled, irregular, mirror and nude) from individuals sampled in Hungary (A) and Singapore (B). Numbers in white at the bottom of the bars indicate the number of individuals analyzed from each scale-pattern category. Different letters on top of the bars indicate statistically significant differences (P-value: <0.01, Student's t-test). Images below panel B show a representative picture of pharyngeal teeth for each of the phenotypes analyzed in Singapore. Note the increased trabeculation and the holes in the pharyngeal arches isolated from the nude individual. Panel C: Side-view of a representative pharyngeal arch from a scaled and nude individual is shown.

The association between the scale pattern and the number of pharyngeal teeth was also tested and compared across crosses involving Hungarian and Asian brooders. There was a progressive loss of pharyngeal teeth in parallel with decreasing scale coverage across both groups, but was much more drastic in the latter. For crosses performed in Hungary, the average teeth numbers for scaled and irregular individuals were similar (10 and 9.75, respectively) while the mirrors and nudes had on an average, ∼8 and 6 teeth (Fig. 4A). In pairwise comparisons, all phenotype pairs, except for scaled and irregular, showed significant differences (P<0.01; Student's t-test). For crosses involving koi brooders, the scaled fishes showed an average number of 9.34 teeth (range: 8–10), whereas the average number of teeth for irregular and mirror individuals was similar (7) and much smaller (<1) for the nudes (Fig. 4B). Almost 70% of the nudes entirely lacked teeth, while the rest of them had between 1–4 teeth only (Fig. 4B). In pairwise comparisons, all phenotype pairs, except for irregular and mirror, showed significant differences (P<0.005; Student's t-test) in their teeth numbers, providing a convincing proof that tooth loss extends to the irregular and mirror categories, not just the nudes as described by Kirpichnikov (Table 8 of [13]). In addition to the loss of teeth, the nudes also displayed a distinct weakening and thinning of the 5-ceratobranchial arch, resulting in its reduction from a three dimensional structure observed in most phenotypes into a thin, boomerang-shaped object (Fig. 4B&C).

Thus, both the fin and teeth defects showed a correlation with the number of scales. However, the latter correlation was much tighter than the former. For instance, individuals classified as nudes exhibited many types of phenotypic variations, which could be broadly subdivided into three sub-groups. Group I (nude1) included those which had at least few scales present either below the dorsal fin or in a randomly distributed manner. In addition, though nudes showed varying degrees of fin loss and/or reduction, in this particular group the loss of fins was not absolute (Fig. 1G). The second group (nude2) invariably lacked scales and almost all the fins were either reduced or absent. In addition, few individuals in this group showed mild body deformity (Fig. 1H). The third group displayed the most extreme phenotype with not only an absolute loss of scales but also of the fins. Also, these individuals displayed severe growth retardation as well as a deformed body shape (nude3, Fig. 1I). While counting the number of teeth in nudes, it was apparent that only those belonging to the nude2 and nude3 categories showed a complete absence of teeth, whereas all those which showed the presence of 1–4 teeth belonged to the nude1 category.

## Discussion

### The loss/distortion of fins and loss of pharyngeal teeth phenotypes are not restricted to the nudes

Nearly a century ago, Rudzinsky [17], [18] described the first set of data on the genetic regulation of scale pattern formation in common carp. Later, Kirpichnikov and Balkashina [19], [25] added more details that eventually led to a complete model [13], [14] that proposed existence of two loci and four alleles, the combination of which resulted in four major phenotypes (listed in the order of decreasing scale cover): fully scaled (wild type), linear, scattered (including mirrors and mirrors with a large number of large extra scales) and nude. In addition to the four major phenotypes, several sub-types were also described [13], [14] as potential deviations from linear or mirror with extra number of scales, but their exact relationship to the main phenotypes was not determined clearly.

Kirpichnikov and his colleagues proposed that there are two genes (S and n; [13], [14]) responsible for scale pattern formation. The homozygous mutant genotype (ss) for the first locus results in partial loss of scales and produces the classical mirror phenotype in carps. The mutation of the second gene (N), when inherited together with two mutant ‘s’ alleles (ssNn) results in the complete absence of scales. We think that ‘s’ and ‘N’ would likely work in a concerted way to regulate the overall expression of the downstream targets or of genes regulated by the action of the two gene products. Thus, the combination of the variable effects from these two genes would result in frequent appearance of intermediate phenotypes in addition to the major ones. Similar phenotypes with large non-overlapping scales were observed in carps with SssNnn genotype generated by triploidization of the eggs from a scaled and nude brooder, presumably due to incomplete dominance of the ‘N’ allele over two wild type ‘n’ alleles [47]. Moreover, triploid nude carps with sssNnn genotype showed less severe phenotypic effects (reduced scale cover and number of anal fin rays) than their diploid counterparts (ssNn; [48]). We argue that instead of removing such sub-types from the system and labeling them as aberrations, they should be included, as their analysis will help us to gain better understanding of this complex situation. Accordingly, we have sub-divided Kirpichnikov's scattered phenotype into two sub-types, and followed their inheritance in several crosses.

The level of tooth loss found in the nudes analyzed by us was more severe in the offspring of Hungarian (ca. 6) and especially in the Singaporean (<1) crosses compared to that observed by Kirpichnikov (7.4; Table 8 of [13]; whereas the values for scaled were 10, 9.34 and 9.22, respectively). The fact that we observed significant reduction of teeth numbers in the mirrors and in the Singaporean irregular individuals, gives an indication that this process might be more complicated than originally thought of. Some of these scattered individuals might carry different ‘N’ alleles with milder effects on the scales, but with the ability to cause reduction in the teeth count.

**Figure 5:**
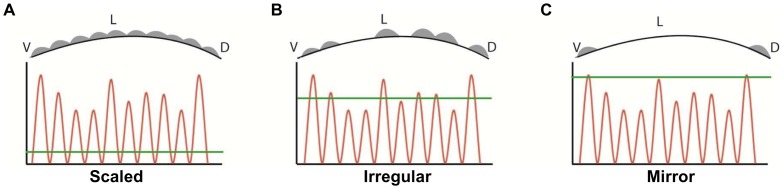
Our working hypothesis showing the rheostat-like action of mutations to the level of signals probably from multiple pathways. An uneven signal level at various locations combined with a gradually decreasing signal threshold in different allelic combinations (plus potential effect from additional modifier genes) might result in the scale pattern phenotypes described in the paper. An imaginary dorso-ventral cross-section shown in the top panel in each case (D: dorsal, L: lateral and V: ventral) shows the typical locations of preferential scale formation on the body surface while the green line indicates the threshold for variants of scaled (A), irregular (B), and mirror carps (C). (An alternate scenario for this hypothesis; stable threshold with decreasing signal intensity from scaled to nude would produce the same outcome.).

Based on the results, we propose that the increased number of scales in the irregular sub-type is the result of an elevated level of expression of genes involved compared to classical mirrors and nudes. This level is higher than that in the mirrors, resulting in the formation of scales at many locations over the body surface, but lower than those that are required for the formation of the wild type pattern. For instance, Eda signaling has been found to exert an effect on scale numbers in zebrafish [36], [37] and teeth number in mouse [41], [49] and in both cases, a dose-dependent effect has been established. Thus, we would like to propose a rheostat-like model – an extension of one suggested earlier by Harris [36], [37] -where the completeness of scale pattern and the formation of fins and teeth are dependent on the overall level of signal (probably through a concerted action of multiple pathways) at the locations where scales are formed. According to this model, although the two genes proposed by Kirpichnikov (S and n; [13], [14]) would be located on two different chromosomes, they would not be fully independent functionally, as they would act along the same pathway(s) regulating the overall level of signaling and thereby the activity of genes regulated by the action of these two gene products (Fig. 5). There are two ways how the gradual loss of signal intensity can be achieved: a) decreasing signals with stable threshold in all phenotypes; and b) steady signal and increasing threshold from scaled to nudes (as shown in Fig. 5). The current state of our knowledge would not allow us to decide, which one of the two is the more likely scenario here.

We do not know the reason why the scales in the irregular sub-type are often bigger and why they aren't arranged in the tight, partially overlapping order as those on the fully scaled wild types are. There might be a temporal increase in the level of one of the signals in these individuals during scale formation that results in the fusion of their precursor cells. Additional research would be needed to find an explanation for this phenotype.

### Unexpected survival rates and proportion of scale pattern phenotypes in the offspring of Hungarian nudes/linears might indicate the presence of a new mutant allele(s)

When two Hungarian brooders carrying the proposed ‘N’ allele were crossed, no lethality was observed among the offspring (Fig. 2). Also, the distortions and losses of fins (Fig. 3) as well as severely reduced pharyngeal teeth counts (Fig. 4B) often detected in Asian nudes, were not observed in most of their Hungarian counterparts. In addition, several crosses involving parents with full or partial Hungarian origin showed unexpected ratios of scale pattern phenotypes, including i) severely reduced proportion (or even complete absence) of nudes from nude × mirror and nude × irregular croses; ii) complete absence of scaled and iii) unexpected proportion of nudes in linear × nude crosses. The earlier study describing the ‘s’ gene found two variants with differential strength of missense alleles in the kinase domain of Fgfr1a1 [24]. In a similar way, our observations seem to indicate that either the Hungarian brooders tested might contain a new mutant ‘s’ allele with stronger effects not described earlier and/or a mutant ‘N’ allele with a milder effect on scale pattern, pharyngeal teeth and fins than the ones described earlier by Kirpichnikov [13], [14]. The (near) complete lack of nude offspring from the two nude × mirror crosses could be produced by a cross between an ordinary mirror and one that carries two hitherto unknown, strong ‘s’ alleles that cause a complete loss-of-function of the gene product resulting in the disappearance of all scales and as such a nude-like phenotype. The other possibility is the presence of a weak ‘N’ allele in the Hungarian nudes that causes the loss of scales, but not the lethality in homozygotes, and it has limited, if any, effect on teeth and fin formation. If we were to assume that the Hungarian nudes all carry the NN genotype, this could potentially explain the vastly reduced proportion of nude offspring produced by the Hungarian nude × mirrors and linear × nude crosses.

At the same time, the Asian ‘N’ allele carried by the koi nudes (and some of their offspring) exerted strong, lasting negative effects on the formation of all three structures. In fact, the cummulative effects of the strong ‘N’ allele are so pronounced that those nude individuals which survive early development are often not able to swim properly and exhibit a distorted body shape either due to skeletal deformations or as a consequence of the lack of fins. When such mutants are grown together with their unaffected (i.e. mirror, irregular, linear or fully scaled) siblings in larger tanks, most of them disappear during the first two months as they lose out in competition for food and get cannibalized by their stronger kins (our unpublished observation). Therefore, in order to save them and analyze them, we had to separate them from the rest and grow them separately. As most previous studies were based on carps grown in ponds from very early developmental stages, it is not surprising that such severely distorted nude individuals have not been described earlier.

At the moment, we do not know the extent these unusual phenotypes (and the proposed underlying mutant alleles) are distributed in the European and possibly Asian populations and stocks. Koi carps are known to be inbred, but strictly controlled, thus individuals showing partial or full scale loss are regularly removed from most stocks.

### The effects of the ‘N’ allele might be dependent on location and developmental timing

Loss or reduction of dorsal fin has been documented from a number of other fish species (see e.g. [50], [51], [52], [53]), especially those under intensive culture. The phenotype is called ‘saddleback’, it is characterized by entirely missing or severely distorted dorsal fins, often together with fusion of some of the vertebrae. It was first described in blue tilapia as a genetically inherited trait, caused by a dominant, lethal mutation [54]. A similar phenotype with complete loss of dorsal fin was identified in goldfish and called the “egg fish”. Although this mutation does not usually result in scale-loss, its additional phenotypes, including decreased stress resistance and increased sensitivity to infections, make it likely that it affects similar developmental pathways in tilapia, as ‘N’ does in nude carps.

One of the advantages of scale-loss phenotypes is that they reveal preferential locations of scale formation that are not detectable on wild type individuals. The two locations, where scales tend to appear even in the case of severe scale loss are the area above the lateral line (in linears) and that below the dorsal fin (in linears, irregulars, mirrors and some nudes). In case of the former, it seems likely that the increased expression levels of the genes involved are maintained during the period of scale formation, resulting in the formation of a line of scales even when the general signal levels are reduced below the threshold necessary for scale fomation at other locations of the body surface. Such phenotypes have been observed in other cyprinids, including the goldfish (according to pictures found on the internet) and grass carp (see Fig. 3 of [55]) and even in a more distantly related Patagonian species, the naked characin (*Gymnocharacinus bergi*, Steindacher, 1903). In this threatened species, the scales first develop over the whole body surface, later they are re-absorbed with the exception of the area covering the lateral line resulting in a linear phenotype [56]. The situation with the other region is more complicated, as there are individuals with a missing dorsal fin and a line of scale below. There are two potential explanations for such a phenomenon: a) the threshold of gene expression required for fin initiation is higher than that needed for scale formation; or b) the early effect of mutation is stronger than the late one.

In summary, we revisited the classical model of scale pattern inheritance proposed by Kirpichnikov and his colleagues in the 1930s. We began by performing a systematic analysis of crosses involving carps of varying scale patterns. On doing this, we found a new scattered phenotype, called irregular, that can be regarded as a variation of mirror with additional scales providing an incomplete coverage of the body surface. As the irregular phenotype was found consistently in many crosses, we incorporated it into the model by dividing the scattered category into mirror and irregular, instead of regarding it as an aberration as Kirpchnikov did. We also addressed the lack of 25% lethality expected based on Kirpichnikov's original genetic model that was observed in nude × nude and nude × linear crosses performed in Hungary. Further, we studied the correlation between the number of scales with fin defects (absence as well as distortions) and teeth loss. We could observe a clear correlation between fin/teeth loss and scale number with such defects being the strongest in nudes and weakest in irregular. Thus, fin and teeth defects in the common carps analyzed in this study are not restricted to the nudes as reported previously.

### Future outlook

After the first publication on the involvement of genetic mechanisms in scale-loss phenotype [17], [18], it took more than 80 years to figure out the identity of the ‘s’ gene [24]. We are currently working on the identification of the second member of this gene pair by following three parallel routes.

Firstly, we have isolated several key members of the Fgf signaling cascade and genes from those upstream pathways that were shown earlier to communicate with this pathway (see e.g. [57], [58]). Comparative sequence analysis of these cDNAs from nude and mirror sibling groups might allow for the identification of the N gene.

Secondly, we have generated several F2 mapping families by crossing Hungarian and Asian representatives of the species with partial or full scale-loss phenotype. Genetic linkage mapping - that is becoming a routine exercise in common carp (see e.g. [59], [60], [61]) - will reveal the chromosomal location that harbors the gene in question. Comparative bioinformatic analysis of the genes contained in syntenic regions of the sequenced teleost models, especially zebrafish might allow for narrowing down the list of potential candidates. Should that approach fail to identify the mutant gene, a map-based positional cloning can be performed for its identification.

Thirdly, rapidly increasing sequence information from traditional [62] and NGS-based sequencing efforts [63], [64] have already yielded benefits for isolation and characterization of full-length cDNA sequences. One of the short-term benefits of these activities is a publicly available high quality transcriptome [63] allowing for RNAseq-based transcriptomics, a substantial improvement from the current method of choice, the cDNA microarray [65].

According to our hope, parallel application of these three approaches will eventually lead to the identification of the ‘N’ gene and more complete understanding of the complex process of scale pattern formation in cyprinids and possibly other teleosts.

## Supporting Information

File S1 **Typical representatives of the four major scale pattern phenotypes in common carp, as classified by Kirpichnikov.** A) Fully scaled (wild type); B) Scattered; C) Linear; and D) Nude individuals.
(TIF)

File S2 **Information on the 18 brooders used for the crosses analyzed.** Abbreviations: M – male; Fe –female; nu – nude; mi – mirror; irreg – irregular; and li – linear.
(XLS)

File S3 **Pictures of the 18 brooders used for the crosses analyzed.**
(TIF)

File S4 **Our revised classification of common carps based on their scale patterns (an extended version of Kirpichnikov's model).**
(DOCX)

File S5 **Representative examples showing the lack of 25% lethality at hatching expected based on Kirpitchnikov's model in a cross involving two Hungarian nude brooders.** Common carp eggs were stuck to a nylon mesh by taking advantage of their natural stickiness immediately after fertilization. The meshes were immersed into separate Zuger jars and kept there for ∼48 hours. Survival rates were estimated by counting surviving embryos with eye spots versus the opaque ones (empty egg shells). A) Mirror × nude cross (control; no large-scale lethality was expected); B) Nude X nude cross (25% of the offspring were expected to die due to their NN genotype) (See Fig. 2 for statistical analysis of several crosses.).
(TIF)

File S6 **Association between the level of scale loss and fin defects in irregular, mirror and nude phenotypes shown in relation to the fin-type.** The percentage of distorted/absent fins is shown along side each fin-type. A (absent), S (stunted), R (reduced) and SLR (slightly reduced). n = 1,341 (irregular), 383 (mirror) and 370 (nude).
(TIF)
