# Supplementary figures and images for: Disappearing Scales in Carps: Re-Visiting Kirpichnikov's Model on the Genetics of Scale Pattern Formation
Source: PLoS One. 2013 Dec 30;8(12):e83327. doi: 10.1371/journal.pone.0083327 (PMC3875451; doi:10.1371/journal.pone.0083327)

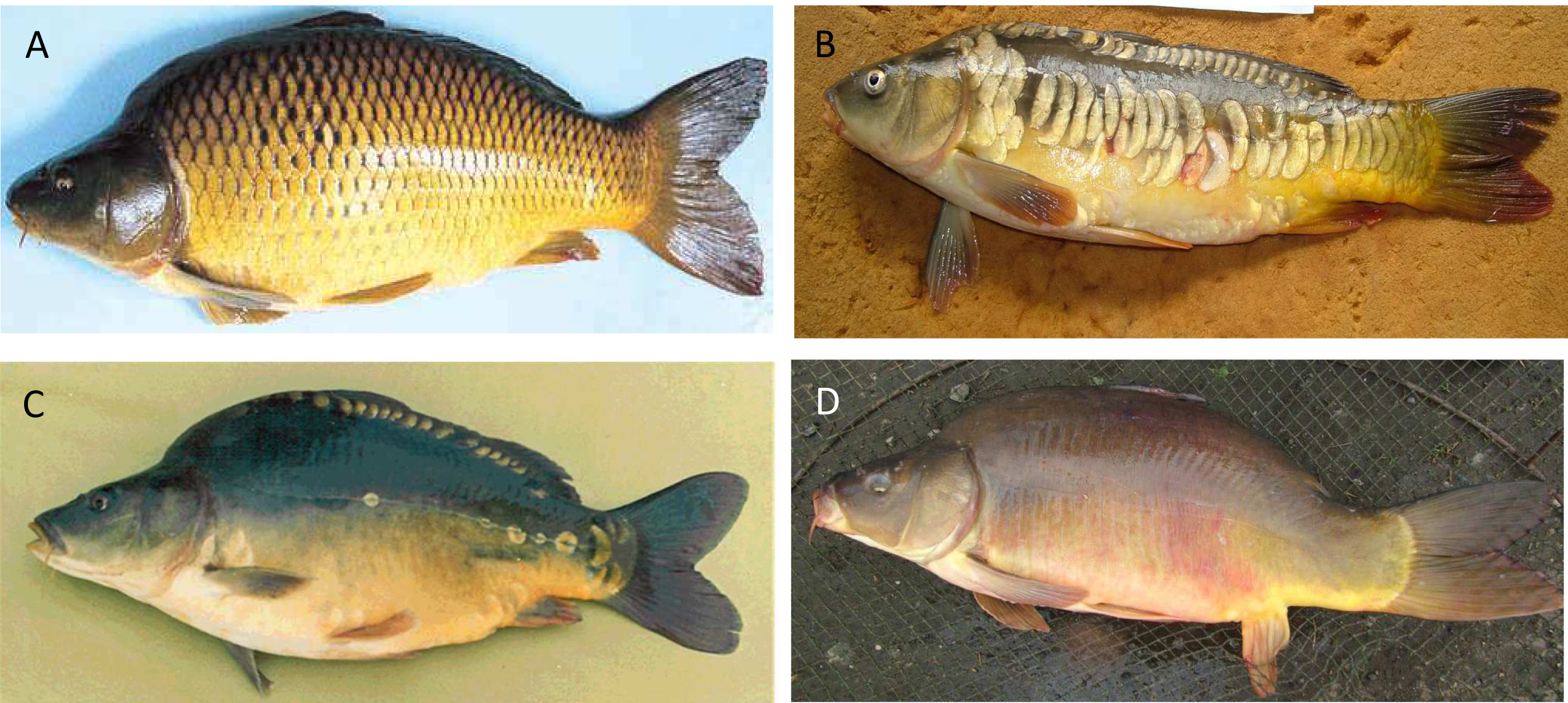

Supplement: File S1 — Typical representatives of the four major scale pattern phenotypes in common carp, as classified by Kirpichnikov. A) Fully scaled (wild type); B) Scattered; C) Linear; and D) Nude individuals. (TIF) [file pone.0083327.s001.tif]

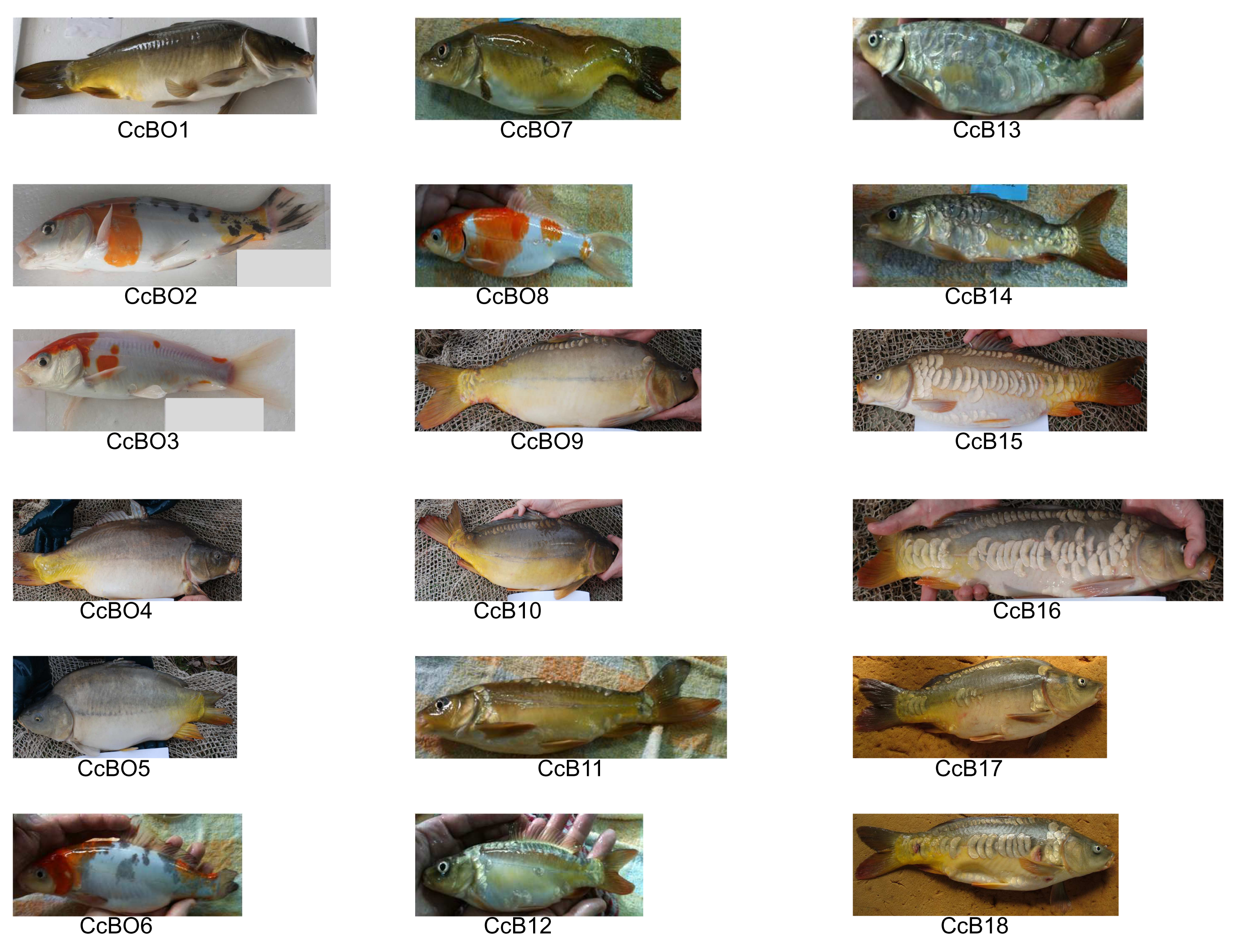

Supplement: File S3 — Pictures of the 18 brooders used for the crosses analyzed. (TIF) [file pone.0083327.s003.tif]

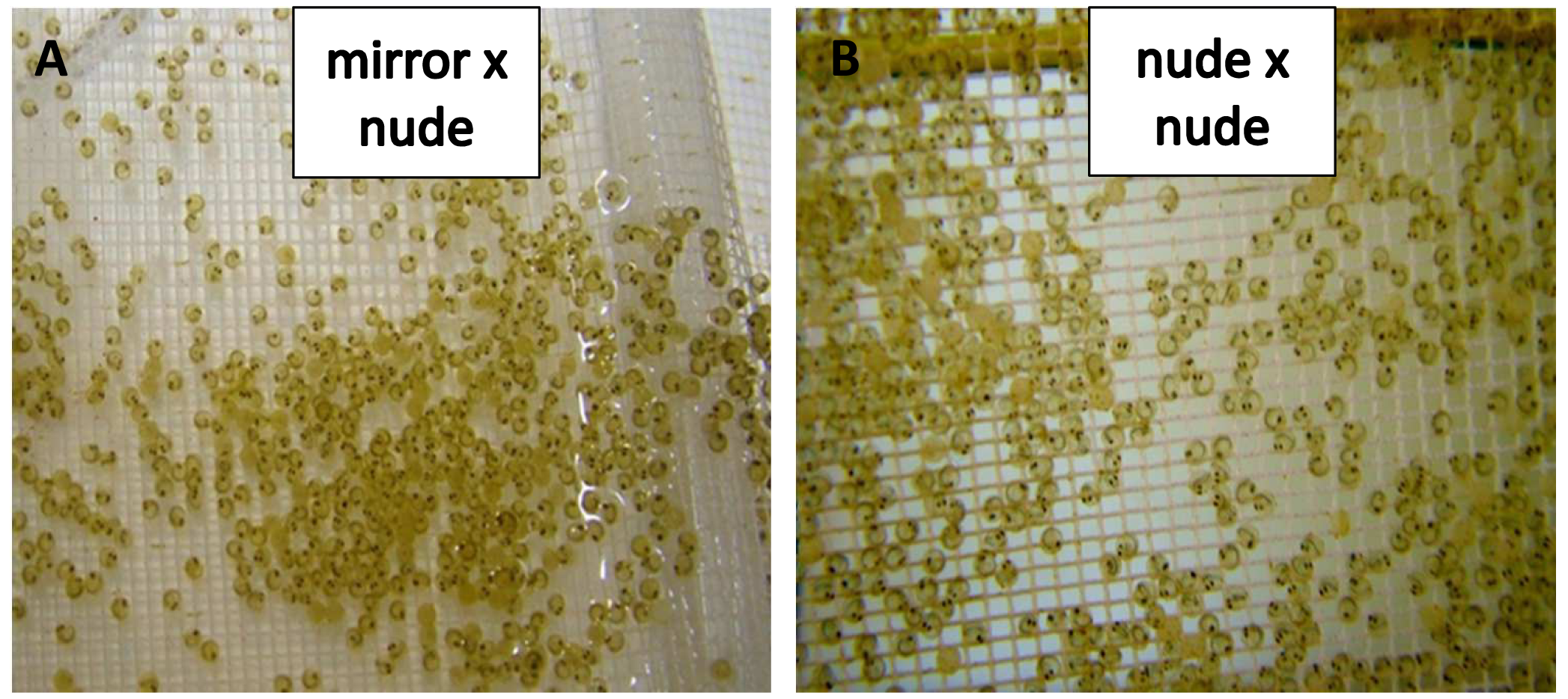

Supplement: File S5 — Representative examples showing the lack of 25% lethality at hatching expected based on Kirpitchnikov's model in a cross involving two Hungarian nude brooders. Common carp eggs were stuck to a nylon mesh by taking advantage of their natural stickiness immediately after fertilization. The meshes were immersed into separate Zuger jars and kept there for ∼48 hours. Survival rates were estimated by counting surviving embryos with eye spots versus the opaque ones (empty egg shells). A) Mirror × nude cross (control; no large-scale lethality was expected); B) Nude X nude cross (25% of the offspring were expected to die due to their NN genotype) (See Fig. 2 for statistical analysis of several crosses.). (TIF) [file pone.0083327.s005.tif]

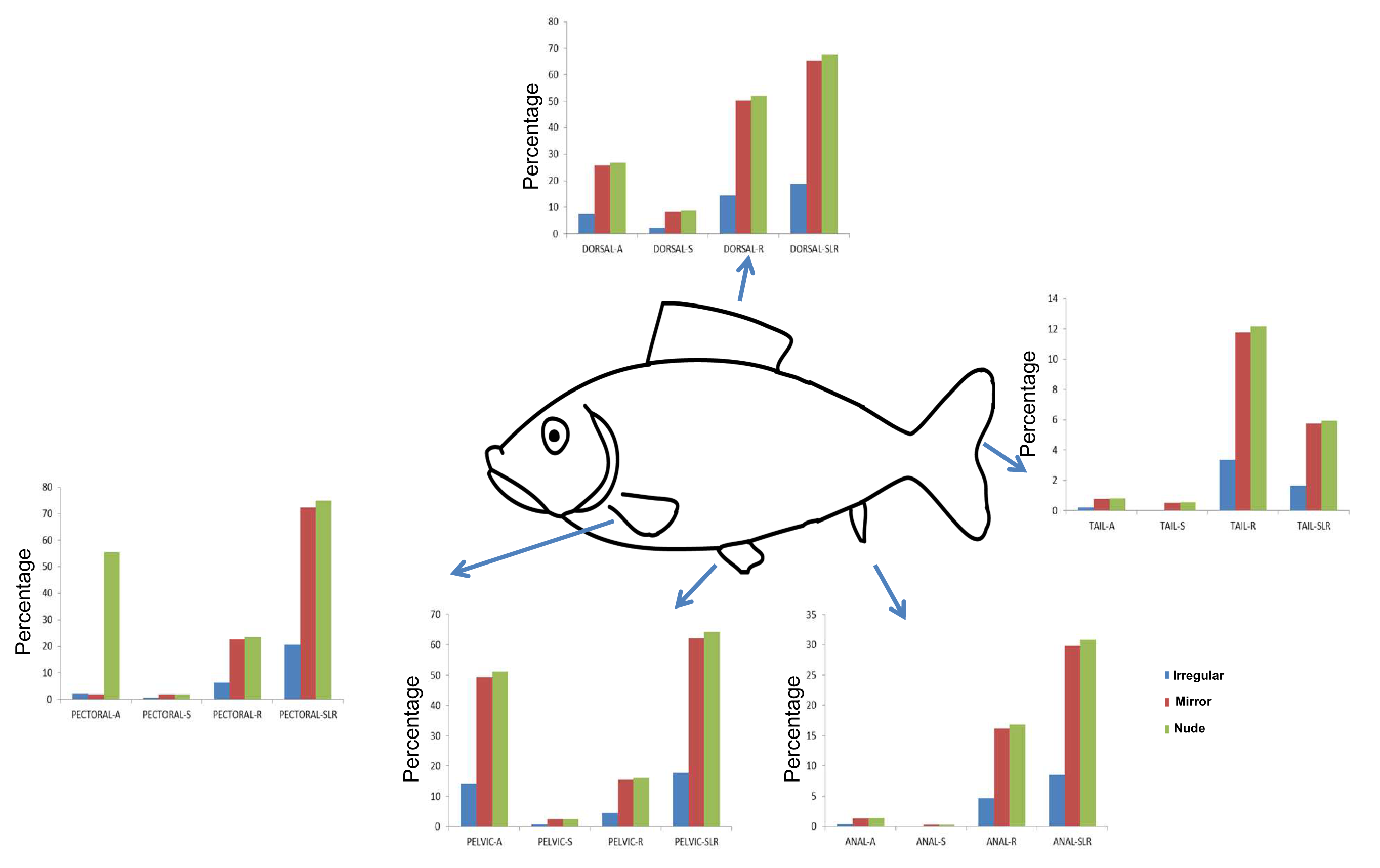

Supplement: File S6 — Association between the level of scale loss and fin defects in irregular, mirror and nude phenotypes shown in relation to the fin-type. The percentage of distorted/absent fins is shown along side each fin-type. A (absent), S (stunted), R (reduced) and SLR (slightly reduced). n = 1,341 (irregular), 383 (mirror) and 370 (nude). (TIF) [file pone.0083327.s006.tif]
