# Supplementary material for: Disappearing Scales in Carps: Re-Visiting Kirpichnikov's Model on the Genetics of Scale Pattern Formation
Source: PLoS One. 2013 Dec 30;8(12):e83327. doi: 10.1371/journal.pone.0083327 (PMC3875451; doi:10.1371/journal.pone.0083327)
Supplement: File S4 — Our revised classification of common carps based on their scale patterns (an extended version of Kirpichnikov's model). (DOCX) [file pone.0083327.s004.docx]

**Supplementary File S4:** Our revised classification of common carps based on their scale patterns (an extended version of Kirpichnikov’s system)

**Scaled (Sc):**  The whole body is covered with regularly arranged scales of very similar size. Every scale partially covers the one located behind it. This is the wild-type pattern.

**Irregular (Ir)**: The body surface has more scales than a mirror. Also, even though there may be individuals with most of the body covered with scales, the scales do not overlap or even reach each other thus leaving the skin exposed among them.

**Linear (Li):** There is a clearly defined line of a uniform set of scales below the dorsal fin and over the lateral line The line(s) might be incomplete. In addition, a lesser number of scales can be found scattered over the body surface.

**Mirror (Mi)**: There is a row of scales (sometimes incomplete) below the dorsal fin, and occasionally another row above the belly (this has a higher probability of being incomplete compared to the former). In addition to these, there might be other scales scattered over the body, especially in the tail region. There is no uniform line of scales over the lateral line and the majority of the body surface is scaleless.

**Nude** **(Nu)**: The scales are either totally absent or very few scales are present (can be randomly distributed but many times are seen as an incomplete line of scales below the dorsal fin). This phenotype is always accompanied by fin and/or teeth defects.
